# Effect of zinc supplementation on glycemic biomarkers: an umbrella of interventional meta-analyses

**DOI:** 10.1186/s13098-024-01366-0

**Published:** 2024-06-08

**Authors:** Maziar Daneshvar, Mohammad Ghaheri, Diba Safarzadeh, Fatemeh Karimi, Parisa Adib-Hajbagheri, Mohadese Ahmadzade, Amir Haedi

**Affiliations:** 1 Tehran University of Medical Science, Tehran, Iran; 2 Student Research Committee, Alborz University of Medical Sciences, Karaj, Iran; 3 Vocational School of Health Service, Near East University, Nicosia, Cyprus; 4 Department of Nutrition, School of Medicine, Hamadan University of Medical Sciences, Hamadan, Iran; 5 Department of Cardiology, Chamran Cardiovascular Research Education Hospital, Isfahan University of Medical Sciences, Isfahan, Iran; 6 Department of Urology, Shahid Labbafinejad Medical Center, Urology and Nephrology Research Center, Shahid Beheshti University of Medical Sciences, Tehran, Iran; 7 Student Research Committee, Faculty of Nutrition and Food Science, Tabriz University of Medical Sciences, Tabriz, Iran

**Keywords:** Zinc supplementation, Glycemic status, Type 2 diabetes, Meta-analysis

## Abstract

**Background:**

Several studies have evaluated the effects of zinc supplementation on glycemic biomarkers in humans and have demonstrated varying results. We systematically evaluated the literature and performed an umbrella meta-analysis of the effects of zinc supplementation on type 2 diabetes biomarkers.

**Methods:**

A comprehensive literature search was conducted in the following databases; PubMed, Embase, Embase, Cochrane Library, Scopus, and Web of Science for studies published up to March 10, 2024.

**Results:**

Zinc supplementation was effective in reducing serum FBS (WMD: − 13.58, 95% CI: − 17.38, − 9.77; p < 0.001; SMD: − 0.52, 95% CI − 0.79, − 0.25; p =  < 0.001), insulin (SMD: − 0.67, 95% CI − 0.96, − 0.38; p < 0.001), HOMA-IR levels (WMD − 0.52, 95% CI − 0.66, − 0.38; p < 0.001; SMD: − 0.78, 95% CI − 1.02, − 0.42; p < 0.001), and HbA1c (WMD: − 0.35, 95% CI − 0.43, − 0.27; p < 0.001).

**Conclusion:**

Zinc supplementation significantly reduced FBS, HOMA-IR, insulin and HbA1c. These findings suggest that zinc is potentially an effective complementary intervention to improve type 2 diabetes biomarkers.

**Supplementary Information:**

The online version contains supplementary material available at 10.1186/s13098-024-01366-0.

## Introduction

Considering the increasing financial burden caused by type 2 diabetes and its associated comorbidities on the health system ($306.6 billion in direct medical costs in the US), attention to more effective treatments for glycemic disorders is increasing [1]. The effect of different micronutrients on type 2 diabetes as one of the most common non-communicable diseases [6.28% of the world’s population [2]] has been investigated in various studies [3]. Different nutritional strategies have been proposed to manage type 2 diabetes including body weight loss, proper macronutrient distribution, choosing food sources from a low glycemic index, consuming fiber intake > 40 g/d, and moderate intake of free sugars [4]. Among the myriad of dietary components, zinc has emerged as a micronutrient of paramount importance, playing a pivotal role in various physiological processes, including immune function, DNA synthesis, and cellular metabolism [5]. Zinc, an essential trace element, is indispensable for the proper functioning of numerous enzymes, transcription factors, and signaling pathways within the human body. Its involvement in insulin metabolism and pancreatic beta-cell function places it at the epicenter of glucose homeostasis. As an integral cofactor for insulin, zinc facilitates insulin hexamer formation and storage in pancreatic beta cells, influencing the release of insulin in response to fluctuating blood glucose levels [6]. Furthermore, zinc is intricately linked to the regulation of glucose transporter proteins, impacting glucose uptake and utilization in peripheral tissues [7]. The perturbation of these processes due to inadequate zinc levels and zinc deficiency has been implicated in the development of insulin resistance and impaired glucose tolerance [8].

Glycemic biomarkers, encompassing a spectrum of indicators reflecting glucose metabolism, provide invaluable insights into an individual's metabolic health. Fasting blood sugar (FBS), glycosylated hemoglobin (HbA1c), insulin levels, and homeostasis model assessment-estimated insulin resistance (HOMA-IR) stand as sentinel markers, offering clinicians a panoramic view of an individual's glycemic status. Elevations in these biomarkers are not only indicative of impaired glucose metabolism but are also key predictors of diabetes onset and its associated complications. The intricate relationship between zinc status and glycemic biomarkers has prompted a surge of interest in elucidating whether optimizing zinc intake could serve as a modifiable factor in glycemic control and diabetes prevention [9].

To synthesize the existing body of evidence on the relationship between zinc intake and glycemic biomarkers, we employ an umbrella systematic review and meta-analysis methodology. This comprehensive approach enables us to encapsulate a wide array of studies, including systematic reviews and meta-analyses, providing a more nuanced understanding of the topic. By amalgamating data from diverse sources, we aim to discern patterns, identify potential sources of heterogeneity, and derive robust conclusions that transcend individual study limitations. Several meta-analysis studies with conflicting results have been performed [10–17]. Some studies showed beneficial effects of zinc on all studied biomarkers [10, 11]; on the other hand, and non-significant results were shown on FBS [12], HbA1c [12], HOMA-IR [12–15], and insulin [14–17]. Also, different statistical methods have been used in different meta-analyses, which have led to different results. As a result, conducting this study using a single statistical method can lead to a definite conclusion about the anti-hyperglycemic effects of zinc.

## Methods

This research adhered to the PRISMA (Preferred Reporting Items for Systematic Reviews and Meta-Analyses) guidelines to ensure thorough reporting. Our protocol was registered in PROSPERO database (CRD42024543516).

### Literature search

To perform this umbrella meta-analysis investigating the impact of zinc supplementation on glycemic index, a systematic search method was applied. Various databases such as PubMed, Embase, Embase, Cochrane Library, Scopus, and Web of Science were scrutinized up to March 10, 2024 using search terms like ((Zinc OR Zn) AND (insulin OR glucose OR glycemic OR HbA1c OR diabet) combined with Meta-analysis. The search terms employed are included in Supplementary Table 1. Additional research was discovered through manual review of reference lists. This study considered only articles in the English language for inclusion.

### Inclusion and exclusion criteria

The PICOS criteria utilized in this umbrella meta-analysis were outlined as follows: Population/Patients (P) consisted of adults aged 18 years and above who had undergone zinc treatment in any health condition; Intervention (I) specifically focused on the administration of zinc; Comparison (C) involved a control group or placebo; Outcome (O) measured the glycemic biomarkers, which included factors like FBS, HbA1c, insulin, and HOMA-IR; (S) meta-analyses of RCTs. Only meta-analysis studies published in English that investigated the impact of zinc supplementation on the type 2 diabetes biomarkers and provided effect sizes (ES) with corresponding confidence intervals (CI) were considered for inclusion. Moreover, only moderate-to-high quality studies scored by AMSTAR method were included. Original studies, editorials, letters to the editor, studies involving children, as well as those involving pregnant or lactating women, were excluded from consideration.

### Evaluating methodology and quality of evidence

Two researchers independently assessed the methodological quality of the incorporated studies utilizing the AMSTAR2 evaluation tool [18], comprising 16 criteria with response categories such as ‘‘yes,’’ ‘‘partial yes,’’ ‘‘no,’’ or ‘‘no meta-analysis.’’ Any inconsistencies were resolved through mutual consensus or with the assistance of a third author if required. The AMSTAR 2 checklist developed four classifications: ‘‘Critical low quality,’’ ‘‘low quality,’’ ‘‘moderate quality,’’ and ‘‘high quality’’. The GRADE approach was utilized to evaluate the studies, which consisted of five factors including, risk of bias, consistency of results, directness, precision, and potential for publication bias. The evidence is classified into four categories: high, moderate, low, or very low [19].

### Study selection and data extraction

Two reviewers independently conducted the screening of studies and extracted data from the identified ones using predetermined criteria. This involved details such as the author's name, year of publication, sample size, study location, dosage and duration of zinc supplementation, as well as, effect sizes (ESs) ((weighted mean difference (WMD), and standardized mean difference (SMD)) and confidence intervals (CI) for glycemic biomarkers.

### Data synthesis and statistical analysis

ESs and CIs were employed to calculate the overall effect. The analysis was conducted separately for SMD and WMD due to their natural differences. Heterogeneity was assessed using Cochran’s Q test and *I*^2^ statistics. If the *I*^2^-value exceeded 50% or the Q-test resulted in a p-value below 0.1, significant between-study heterogeneity was acknowledged. To address this, the random-effects model was utilized. Subgroup analysis based on predetermined variables such as intervention duration, health condition, supplementation dosage, and mean age helped identify potential sources of heterogeneity. Sensitivity analysis was performed to assess the impact of excluding a single study on the combined effect size. Egger’s and Begg’s tests were utilized to examine small-study effects. Publication bias was evaluated through visual examination of funnel plots, and if detected, a trim and fill test was subsequently conducted. The meta-analysis was conducted using STATA (version 16, Stata Corporation, College Station, TX, United States). A significance level of p < 0.05 was set for this study.

## Result

### Selected studies and systematic review

**Fig. 1:**
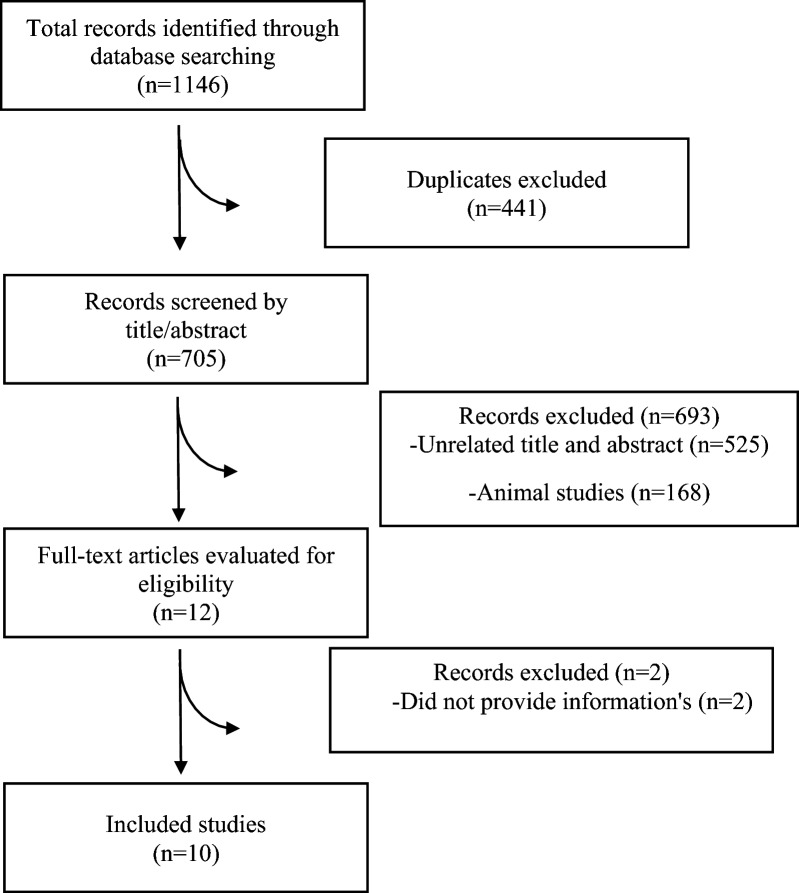
Flow diagram of study selection

The PRISMA flowchart illustrating the process of literature search is presented in Fig. 1. Initially, 1146 articles were identified through electronic database searches, with 441 duplicates. Upon reviewing the titles and abstracts of the remaining 705 studies, 693 articles did not meet the inclusion criteria and were consequently excluded from further analysis. Ultimately, 10 meta-analyses (13 ESs) published between 2012 and 2023 met the requirements for inclusion in the umbrella review. Table 1 outlines the characteristics of these included meta-analyses. The average administered dosage of zinc across the studies ranged between 18.04 and 210 mg/day. The duration of zinc supplementation varied from 6.6 to 18 weeks. The studies were conducted across different locations: two in China [10, 20], four in Iran [14–16, 21], one in UK [17], one in USA [12], one in Taiwan [22], and one in Sri Lanka [23]. One included study in the meta-analysis by Jayawardena et al. reported that two cases of mild abdominal pain in patients receiving Zinc sulfate 660 mg/day for 12 weeks [24].

**Table 1:** Study characteristics of included studies

Citation (First author et al. Year)^a^	RCTs included in study	Sample size/Gender	Mean age /Duration (Wk)	Study population	Zinc dosage (mg)	Publication bias Egger’s test	Quality Assessment (Number of included study)/Grade approach	Main Outcome/Heterogeneity measures
Khazdouz et al. [14] Iran	15	1141/ Both	38.4–55.65/7–12week	T2DM—Obesity—Prediabetes -GDM	37.4–104.9	P = 0.07	Cochrane risk of bias 2/15 high quality No	FBS↓ I^2^; 88.3, Q test;119.78, p < 0.001 A1c↓ I^2^; 91.4, Q test;105.20, p < 0.001 HOMA-IR ↔ I^2^; 95.6, Q test;135.23, p < 0.001 insulin ↔ I^2^; 91.4, Q test;105.20, p < 0.001
Li et al. [10] China	5	263/ Female	NR	GDM	NR/zinc gluconate	NR	Jada scores 3/5 high quality No	FBS↓ I^2^; 0.0, Q test, p = 0.59 HOMA-IR↓ I^2^; 0.0, p = 0.91 insulin↓ I^2^; 0.0, p = 0.96
Ghaedi et al. [13]. Iran	17	1442/ Both	46.93- 55.26/9–16 week	T2DM-DN-Diabetic foot ulcer-T2DM + microalbuminuria	40–86.17/zinc sulfate, zinc gluconate	P ˃ 0.05	Cochrane risk of bias 4/17 high quality Moderate to high certainty	FBS↓ I^2^; 93.57, p < 0.001 A1c↓ I^2^; 80.54, p < 0.001 HOMA-IR ↔ I^2^; 82.26, p < 0.001 insulin ↔ I^2^; 81.84, p < 0.001
Jayawardena et al. [23] Sri Lanka	12	579/Both	50.93- 51.93/12–14 week	T2DM	117.75–210/Zn acetate, zinc sulfate, zinc gluconate	NR	NR No	FBS↓ I^2^; 99.0, p < 0.001 A1c↓ I^2^; 95.0, p < 0.001
Nazari et al. [15] Iran	23	1315/Both	54.55- 68.62/12–18 week	Prediabetes and T2DM	36.6–114/Zinc	P ˃ 0.05	Cochrane risk of bias 12/23 high quality Very low to low certainty	FBS↓ I^2^; 94.3, p < 0.001 A1c↓ I^2^; 91.5, p < 0.001 HOMA-IR ↔ I^2^; 73.4, p < 0.001 insulin ↔ I^2^; 82.3, p < 0.001
Nazari et al. [16] Iran	34	1969/Both	43.89- 54.27/8–14 week	T2DM- T1DM- Obesity- Healthy—Competitive Athletes- Pregnant Women with Impaired Glucose Tolerance—GDM- PCOS- Diabetic foot ulcer—NAFLD	34.18–70.1 /Zinc	P ˃ 0.05	Cochrane risk of bias 24/34 high quality Very low to low certainty	FBS↓ I^2^; 92.6, p < 0.001 A1c↓ I^2^; 91.0, p < 0.001 HOMA-IR↓ I^2^; 72.2, p < 0.001 insulin ↔ I^2^; 78.4, p < 0.001
Pompano (A) et al., 2018. USA	6	368/ Both	29.9–50/10–19 week	T2DM- Healthy—Prediabetes	16.1- 20/ Zinc	P˃0.05	Cochrane risk of bias 6/6 high quality No	FBS↓ I^2^; 96.0, p < 0.001 A1c ↔ I^2^; 0.0, p = 0.78 HOMA-IR↓ I^2^; 62.0, p = 0.07
Pompano (B) et al. 2018. USA	14	1039/ Both	23.5- 56.16/8–10 week	T2DM- Healthy—Prediabetes-PCOS- Pregnant impaired glucose tolerance	37.77- 107.85/ Zinc	P˃0.05	Cochrane risk of bias 14/14 high quality No	FBS ↔ I^2^; 91.0, p < 0.001 A1c↓ I^2^; 88.0, p < 0.001 HOMA-IR↓ I^2^; 55.0, p = 0.02
Pompano (C) et al. USA	11	704/ Both	29.25- 53.73/6.5–7.5 week	T2DM- Healthy- Prediabetes-PCOS-GDM	33.75- 188/ Zinc	P˃0.05	Cochrane risk of bias 11/11 high quality No	FBS↓ I^2^; 93.0, p < 0.001 A1c ↔ I^2^; 36.0, p = 0.19 HOMA-IR↓ I^2^; 61.0, p = 0.009
Pompano (D) et al. [12]. USA	5	703/ Both	52.51–56.43/12–24 week	T2DM- Healthy—Prediabetes-Pregnant impaired glucose tolerance	33.28–37.16/Zinc	P ˃ 0.05	Cochrane risk of bias 5/5 high quality No	FBS↓ I^2^; 71.0, p < 0.001 A1c ↔ I^2^; 89.0, p < 0.001 HOMA-IR ↔ I^2^; 61.0, p = 0.08
Wang et al. [20]. China	36	1139/ Both	21.16- 58.4/10–13week	T2DM with microalbuminuria- Pregnant impaired glucose tolerance—Obesity- GDM	31.88- 140.1 /Zn acetate, zinc sulfate, zinc Gluconate, zinc amino acids	P ˃ 0.05	Jada scores 23/36 high quality No	FBS↓ I^2^; 94.5, p < 0.001 A1c↓ I^2^; 97.6, p < 0.001 HOMA-IR↓ I^2^; 98.6, p < 0.001 insulin ↔ I^2^; 98.1, p < 0.001
Wang et al. [17]. UK	12	392/ Both	47.41–53.85/10–11.5 week	T2DM- Diabetic hemodialysis patients- Diabetes with β-thalassemia major complicated- DN	20.57–41.07/elemental Zn, zinc sulfate, zinc Gluconate	P ˃ 0.05	Cochrane risk of bias 9/12 high quality No	FBS↓ I^2^; 52.91, p = 0.06 A1c↓ I^2^; 65.72, p = 0.02 HOMA-IR↓ I^2^; 76.12, p = 0.01 insulin ↔ I^2^; 74.33, p < 0.001
Yang et al. 2023. Taiwan	12	495/Both	39.11–46.14/8–11 week	Overweight and obese	29.37–35.83/zinc sulfate, zinc Gluconate, Zinc amino chelate	P ˃ 0.05	Cochrane risk of bias 7/12 high quality Low to moderate certainty	FBS↓ I^2^; 83.0, p < 0.001 A1c↓ I2; 71.0, p < 0.001 HOMA-IR↓ I^2^; 0.0, p = 0.6

*NR* not reported, *PCOS* Polycystic ovary syndrome, *NAFLD* nonalcoholic fatty liver disease, *GDM* Gestational Diabetes Mellitus, *T2DM* Type 2 diabetes, *DN* Diabetic nephropathy

^a^All meta-analyses used random effects models

### Methodological quality and quality of evidence

Table 2 displays results from the AMSTAR2 questionnaire, revealing that eight articles were evaluated as high quality, and while two was categorized as moderate quality. The GRADE approach showed that HbA1c had high-quality evidence, but insulin, HOMA-IR, and FBS having moderate-quality evidence (Table 3).

**Table 2:** Results of assess the methodological quality of meta-analysis

Study	Q1	Q2	Q3	Q4	Q5	Q6	Q7	Q8	Q9	Q10	Q11	Q12	Q13	Q14	Q15	Q16	Quality assessment
Khazdouz et al. [14]	No	Partial Yes	Yes	Partial Yes	Yes	Yes	Yes	Yes	Yes	No	Yes	Yes	Yes	Yes	Yes	Yes	High
Li et al. [10]	No	Partial Yes	No	Partial Yes	Yes	Yes	Yes	Yes	Yes	No	Yes	Yes	No	Yes	No	No	Moderate
Ghaedi et al. [13]	Yes	Yes	Yes	Yes	Yes	Yes	Yes	Yes	Yes	Yes	Yes	Yes	Yes	Yes	Yes	Yes	High
Jayawardena et al. [23]	No	Partial Yes	Yes	Partial Yes	Yes	Yes	Yes	Yes	Yes	No	Yes	Yes	Yes	No	Yes	Yes	Moderate
Nazari et al. [15]	No	Partial Yes	Yes	Partial Yes	Yes	Yes	Yes	Yes	Yes	Yes	Yes	Yes	Yes	Yes	Yes	Yes	High
Nazari et al. [16]	No	Yes	Yes	Partial Yes	Yes	Yes	Yes	Yes	Yes	Yes	Yes	Yes	Yes	No	Yes	Yes	High
Pompano et al. [12]	No	Partial Yes	Yes	Partial Yes	Yes	Yes	Yes	Yes	Yes	Yes	Yes	Yes	Yes	Yes	Yes	Yes	High
Wang et al. [20]	No	Partial Yes	Yes	Yes	Yes	Yes	Yes	Yes	Yes	No	Yes	Yes	Yes	Yes	Yes	Yes	High
Wang et al. [17]	No	Partial Yes	Yes	Partial Yes	Yes	Yes	Yes	Yes	Yes	Yes	Yes	Yes	Yes	Yes	Yes	Yes	High
Yang et al. 2023	No	Partial Yes	Yes	Yes	Yes	Yes	Yes	Yes	Yes	No	Yes	Yes	Yes	Yes	Yes	Yes	High

**Table 3:** Summary of findings and quality of evidence assessment using the GRADE approach

Glycemic measures	Summary of findings		Quality of evidence assessment (GRADE)					
	No of patients (effect sizes)	WMD (95% CI)	Risk of bias^b^	Inconsistency ^c^	Indirectness ^d^	Imprecision ^e^	Publication bias^f^	Quality of evidence^g^
FBS	9940 (11)	− 13.58 (− 17.38, − 9.77)	Not Serious	Serious	Not Serious	Not Serious	Not Serious	Moderate
HbA1C (%)	6676 (11)	− 0.35 (− 0.43, − 0.27)	Not Serious	Not Serious	Not Serious	Not Serious	Not Serious	High
HOMA-IR	4958 (10)	− 0.52 (− 0.66, − 0.38)	Not Serious	Not Serious	Not Serious	Serious	Not Serious	Moderate
Insulin	3463 (5)	− 0.16 (− 0.74, 0.43)	Not Serious	Not Serious	Not Serious	Serious	Not Serious	Moderate

*FBS* Fasting blood sugar, *HbA1C* Hemoglobin A1C; *HOMA-IR* Homeostatic Model Assessment for Insulin Resistance

^a^Presented as weighted mean difference (WMD) all outcomes

^b^Risk of bias based on the AMSTAR2 questionnaire

^c^Downgraded if there was a substantial unexplained heterogeneity (*I*^2^ > 50%, *P* < 0.10) that was unexplained by meta-regression or subgroup analyses

^d^Downgraded if there were factors present relating to the participants, interventions, or outcomes that limited the generalizability of the results

^e^Downgraded if the 95% confidence interval (95% CI) crossed the minimally important difference (MID) for benefit or harm. MIDs used for each outcome were: 0.3% for HbA1C, 9 mg/dl for FBS, 1.2 μIU/dl for insulin, 1.77 for HOMA-IR, 3.09 for HOMA-β, and 0.32 for QUICKI

^f^Downgraded if there was an evidence of publication bias using funnel plot that affected overall results detecting by trim and fill analysis

^g^Since all included studies were meta-analysis of randomized controlled trials, the certainty of the evidence was graded as high for all outcomes by default and then downgraded based on prespecified criteria. Quality was graded as high, moderate, low, very low

### The effects of zinc on FBS levels

**Fig. 2:**
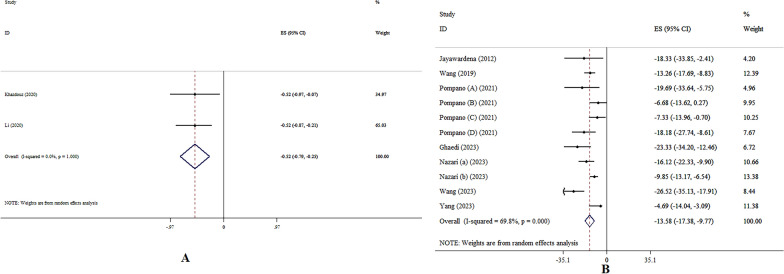
Forest plot detailing effect size and 95% confidence intervals (CIs), the effects of zinc supplementation on FBS levels according to SMD (**A**), and WMD (**B**) analysis

Zinc supplementation resulted in a significant reduction in FBS levels, according to the SMD analysis (SMD: − 0.52, 95% CI − 0.79, − 0.25; p =  < 0.001, *I*^2^ = 0.0%, p = 0.999) (Fig. 2A) [10, 14]. The supplementation of zinc resulted in a significant decrease in FBS levels, according to the WMD analysis (WMD: − 13.58, 95% CI − 17.38, − 9.77; p < 0.001, *I*^2^ = 69.8%, p < 0.001) (Fig. 2B) [12, 15–17, 20–23]. Additionally, the results from subgroup analysis suggested that participant age, dosage, and health conditions were identified as the origins of heterogeneity (Table 4).

**Table 4:** Subgroup analyses for the effects of zinc supplementation on glycemic biomarkers

	NO	WMD (95% CI)^a^	P-within^b^	*I*^2^ (%)^c^	P-heterogeneity^d^
Zinc supplementation on FBS					
Overall	11	− 13.58 (− 17.38, − 9.77)	< 0.001	69.8	< 0.001
Age (year)					
< 50	7	− 11.46 (− 15.92, − 7.91)	< 0.001	73.9	0.001
≥ 50	4	− 17.99 (− 22.50, − 13.49)	< 0.001	0.0	0.734
Intervention duration (week)					
≤ 10	5	− 11.28 (− 17.63, − 4.93)	0.001	81.1	< 0.001
> 10	6	− 15.90 (− 20.80, − 11.00)	< 0.001	51.7	< 0.001
Dosage of zinc (mg/day)					
< 100	8	− 13.22 (-18.44, − 8.20)	< 0.001	76.1	0.674
≥ 100	3	− 14.43 (-17.94, − 10.91)	< 0.001	0.0	< 0.001
Health conditions					
Diabetic	5	−18.51 (− 23.81, − 13.22)	< 0.001	54.6	0.066
Others	5	− 10.37 (− 14.16, − 6.58)	< 0.001	35.2	0.187
Obesity	1	− 4.69 (− 10.16, 0.78)	0.093	0.0	–
Zinc supplementation on HbA1C					
Overall	11	− 0.35 (− 0.43, − 0.27)	< 0.001	0.0	0.573
Age(year)					
< 50	3	− 0.34 (− 0.48, − 0.19)	< 0.001	5.2	0.348
≥ 50	8	− 0.36 (− 0.45, − 0.26)	< 0.001	0.0	0.496
Intervention duration (week)					
≤ 10	4	− 0.26 (-0.40, -0.11)	0.001	0.0	0.532
> 10	7	− 0.39 (-0.48, -0.30)	< 0.001	0.0	0.653
Dosage of zinc (mg/day)					
< 100	6	− 0.37 (− 0.47, − 0.26)	< 0.001	0.0	0.681
≥ 100	5	− 0.34 (− 0.48, − 0.20)	< 0.001	23.4	0.265
Health conditions					
Diabetic	8	− 0.44 (− 0.55, − 0.33)	< 0.001	0.0	0.993
Others	2	− 0.27 (− 0.49, − 0.04)	0.019	62.5	0.103
Obesity	1	− 0.25 (− 0.43, − 0.27)	0.006	–	–
Zinc supplementation on HOMA-IR					
Overall	10	− 0.52 (− 0.66, − 0.38)	< 0.001	15.0	0.305
Age (year)					
< 50	7	− 0.56 (− 0.71, − 0.42)	< 0.001	12.1	0.337
≥ 50	3	− 0.29 (− 0.60, 0.02)	0.070	0.0	0.503
Intervention duration (week)					
≤ 10	5	− 0.60 (− 0.80, − 0.40)	< 0.001	25.1	0.254
> 10	5	− 0.44 (− 0.61, − 0.27)	< 0.001	0.0	0.419
Health conditions					
Diabetic	5	− 0.66 (− 1.13, − 0.18)	0.006	58.7	0.046
Others	4	− 0.51 (− 0.67, − 0.35)	< 0.001	0.0	0.846
Obesity	1	− 0.54 (− 0.78, − 0.30)	< 0.001	–	–
Zinc supplementation on insulin					
Overall	5	− 0.16 (− 0.74, 0.43)	0.597	26.3	0.246
Intervention duration (week)					
≤ 10	3	− 0.48 (− 1.79, 0.83)	0.469	46.6	0.154
> 10	2	− 0.06 (− 0.47, 0.34)	0.756	0.0	0.342
Health conditions					
Diabetic	4	− 0.01 (− 0.79, 0.77)	0.974	33.0	0.214
Others	1	− 0.64 (− 1.70, 0.42)	0.756	–	–

*WMD* weighted mean differences, *CI* confidence interval

^a^Obtained from the Random-effects model

^b^Refers to the mean (95% CI)

^c^Inconsistency, percentage of variation across studies due to heterogeneity

^d^Obtained from the Q-test

### The effects of zinc on HbA1C levels

**Fig. 3:**
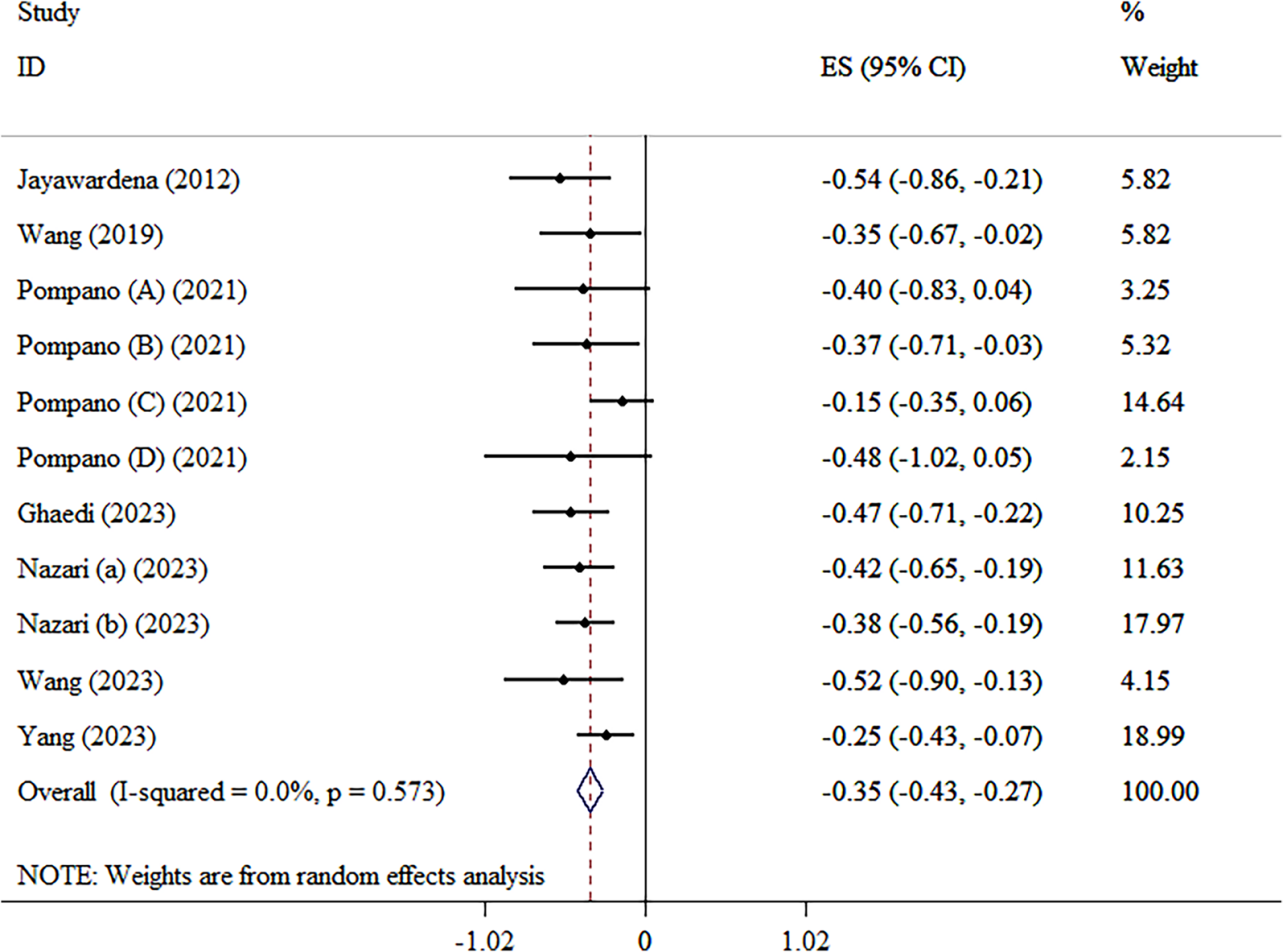
Forest plot detailing mean difference and 95% confidence intervals (CIs) of the effects of zinc supplementation on HbA1C levels according to WMD analysis

The administration of zinc supplements led to a notable reduction in HbA1c levels, according to the WMD analysis (WMD: − 0.35, 95% CI − 0.43, − 0.27; p < 0.001, *I*^2^ = 0.0%, p = 0.57) (Fig. 3) [12, 15–17, 20–23]. The subgroup analysis indicated that the impact of consuming zinc in reducing HbA1c is more noticeable in individuals diagnosed with diabetes (Table 4).

### The effects of zinc on HOMA-IR levels

**Fig. 4:**
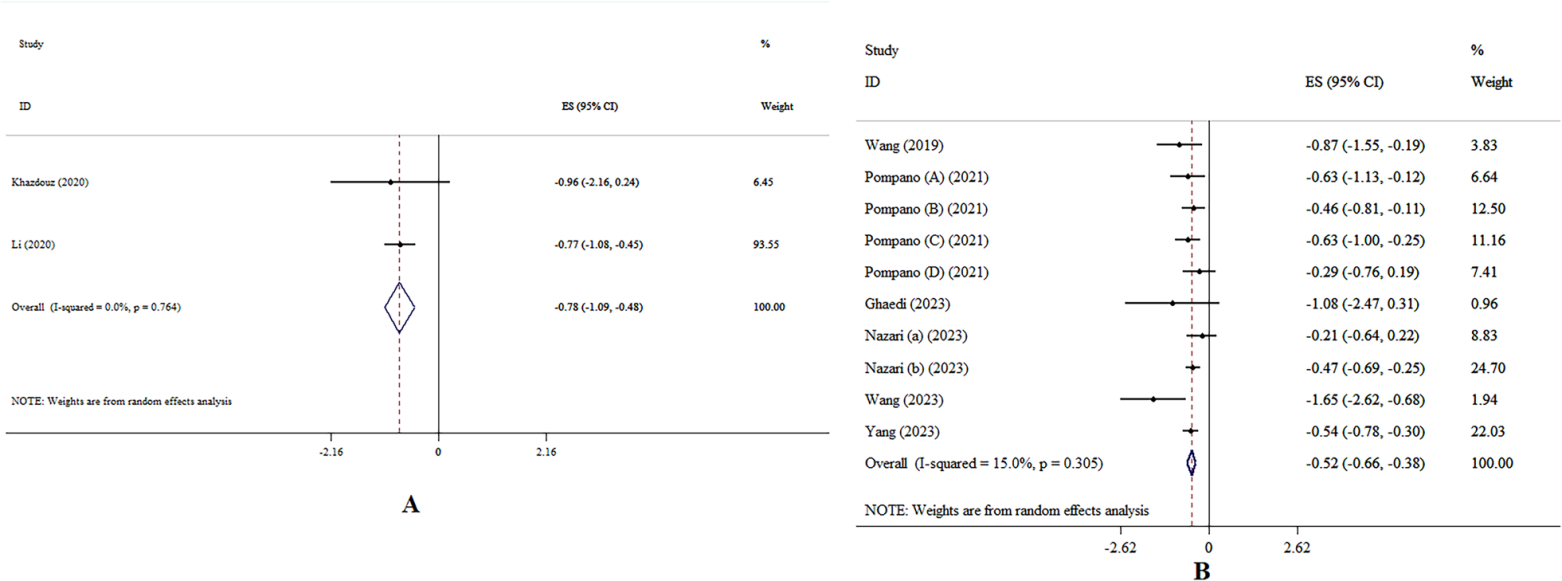
Forest plot detailing effect size and 95% confidence intervals (CIs), the effects of zinc supplementation on HOMA-IR levels according to SMD (**A**), and WMD (**B**) analysis

Zinc supplementation led to a significant decrease in HOMA-IR, according to the SMD analysis (SMD: − 0.78, 95% CI − 1.02, − 0.42; p < 0.001, *I*^2^ = 0.0%, p = 0.53) (Fig. 4A) [10, 14]. The addition of zinc resulted in a noteworthy reduction in HOMA-IR, according to the WMD analysis (WMD: − 0.52, 95% CI − 0.66, − 0.38; p < 0.001, *I*^2^ = 15%, p = 0.30) (Fig. 4B) [12, 15–17, 20–22]. Subgroup analysis showed that the effect of zinc supplementation in reducing A1c is more significant in people with diabetes, age < 50 years, and intervention duration ≤ 10 weeks (Table 4).

### The effects of zinc on insulin levels

**Fig.5:**
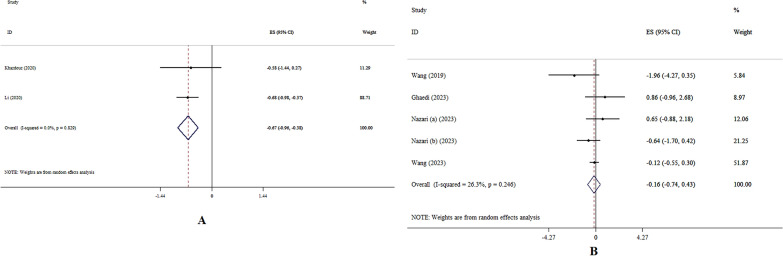
Forest plot detailing effect size and 95% confidence intervals (CIs), the effects of zinc supplementation on insulin levels according to SMD (A) , and WMD (B) analysis

Zinc supplementation significantly decreased serum insulin levels, according to the SMD analysis (SMD: − 0.67, 95% CI − 0.96, − 0.38; p < 0.001, *I*^2^ = 0.0%, p = 0.82) (Fig. 5A) [10, 14]. Administration of zinc supplementation did not lead to a significant decrease in serum insulin levels, according to the WMD analysis (WMD: − 0.16, 95% CI − 0.74, 0.43; p = 0.59, *I*^2^ = 26.3%, p = 0.24) (Fig. 5B) [15–17, 20, 21]. The lack of a significant result in subgroup analyses remained consistent and did not show any significant findings (Table 4).

### Sensitivity analysis and publication *bias*

There were no notable variations detected in the sensitivity analysis concerning each of the examined factors.

Neither Egger’s nor Begg’s tests indicated a small study effect (P˃0.05). The trim and fill method was performed following the uneven distribution of the funnel plots (Supplementary Figs. 1–4), and no imputed study was included for glycemic biomarkers.

## Discussion

The current umbrella systematic review and meta-analysis was conducted with the aim of investigating the effect of zinc supplementation on glycemic biomarkers and the findings showed that zinc supplementation has an improving effect on these biomarkers. Therefore, zinc can be considered as an adjuvant therapy in improving glycemic parameters like other adjuvant therapy agents such as okra [25], probiotic [26], purslane [27], curcumin [28], flaxseed [29], cinnamon [30], and cumin [31]. The rationale of the present study is the existence of meta-analysis studies with conflicting results. Some studies showed beneficial effects of zinc on all studied biomarkers [10, 11]; on the other hand, non-significant results were shown on FBS [12], HbA1c [12], HOMA-IR [12–15], and insulin [14–17]. The reason for the difference in the results of the studies can be related to the study population, the dosage and duration of the supplementation, and the statistical methods used (random effects vs. fixed effects model or WMD vs. SMD calculation). Although the population of the included studies had diseases of different natures, they were similar in one aspect and that is having insulin resistance as one of the causes of their pathogenesis. As a result, the results of these studies were comparable from this point of view. Also, subgroup analysis was performed based on the study population to obtain more accurate results. For a more detailed analysis, we analyzed the studies that reported SMD and the studies that reported WMD separately. Since SMD adjusts results based on SD, in some analyzes results from WMD differed from SMD, which could be due to wide SD range [32]. For example, zinc does not have a positive effect on insulin levels when considering WMD, whereas zinc leads to a significant improvement in insulin when considering SMD. There are different methods and kits with different sensitivities and specificities for measuring the level of biomarkers, which can have different results on same biomarker. Therefore, the SMD report can be more accurate than the WMD, which reports a raw mean difference. However, the results on the effect of zinc on insulin levels should be interpreted with caution. The quality of most of the included studies was acceptable, which makes the results more valid.

**Fig.6:**
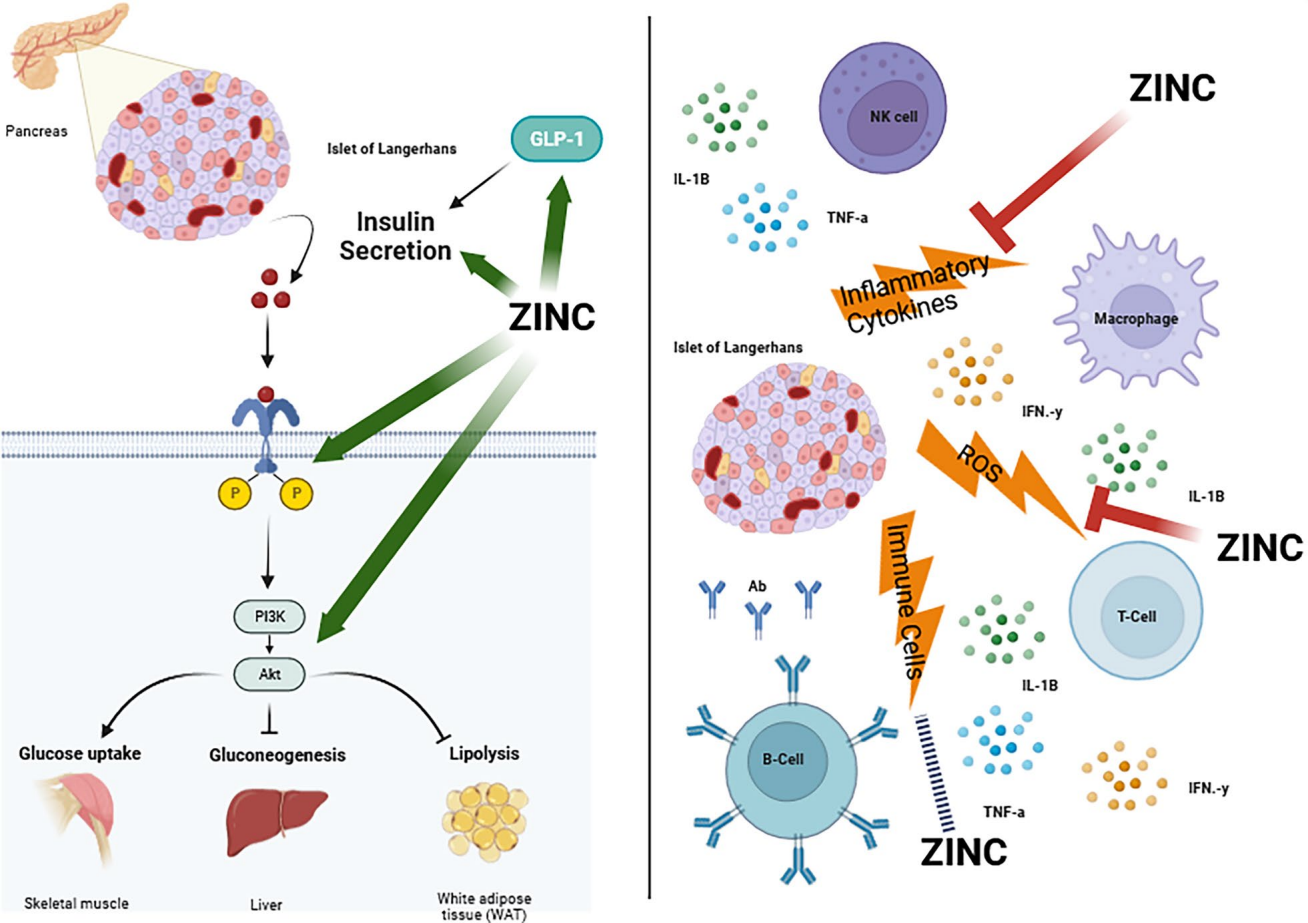
The mechanism of zinc intake on glycemic indices

Subgroup analysis showed that zinc has an improving effect on glycemic indices in both subgroups of age (< 50 and ≥ 50 years), dose (< 100 and ≥ 100 mg/day), duration of supplementation (≤ 10 and > 10 weeks). It must be noted that the Tolerable Upper Intake Level (UL) of zinc for adults is 40 mg/day [33], therefore, high doses should be taken with caution. The side effects of high zinc intake are digestive problems, headaches, decreased concentrations of HDL cholesterol, reduced copper and iron status, and impairment in immunological response [33]. The greatest effect of this supplement was shown in hyperglycemic status in diabetic patients, which seems reasonable. The mechanisms of zinc's anti-hyperglycemic actions are not fully understood, but several potential mechanisms have been proposed. Zinc may activate insulin receptor tyrosine kinase, enhancing insulin sensitivity [34]. This activation can lead to increased glucose uptake by cells, particularly in skeletal muscle and adipose tissue. Moreover, zinc may modulate key components of the insulin signaling pathway, such as phosphoinositide 3-kinase (PI3K) and protein kinase B (Akt), promoting glucose uptake and utilization [35]. Zinc is concentrated in pancreatic β-cells, where it is co-released with insulin. It has been proposed that zinc may play a role in insulin granule formation and insulin secretion [36]. In addition, zinc has antioxidant properties [37, 38], and by protecting β-cells from oxidative stress, it may help maintain their function and prevent apoptosis. Regarding gluconeogenesis, zinc may inhibit the enzymes involved in gluconeogenesis [39], the process by which the liver produces glucose. By limiting glucose production, zinc can contribute to maintaining normal blood glucose levels. In addition, it has been found that zinc may facilitate glucose uptake by cells, similar to its insulin-sensitizing effects [40]. This could contribute to lowering blood glucose levels. Another anti-hyperglycemic effect of zinc is related to its anti-inflammatory [41, 42] and immunomodulatory effects [43, 44]. Chronic inflammation and immune imbalance are associated with insulin resistance [45]. Apart from its effect on insulin, some studies have suggested that zinc may enhance GLP-1 (Glucagon-Like Peptide-1) secretion, contributing to improved glucose homeostasis [46]. GLP-1 is an incretin hormone that stimulates insulin secretion and inhibits glucagon release. It's important to note that while these mechanisms are supported by some experimental and clinical studies, the exact role of zinc in glucose metabolism is complex and may involve interactions with other factors (Fig. 6). Moreover, the optimal dosage of zinc for anti-hyperglycemic effects and potential side effects need further investigation. Three included studies performed dose–response analysis. Two of three studies found that the association between zinc and glycemic parameters was in a duration dependent manner not dose dependent [15, 16]. Ghaedi et al. reported that the association between zinc and HbA1c and FBS was dose-dependent [13]; so that, the more improving effect was shown in 40 mg/day dose. This study was conducted on diabetic subjects; therefore, dose–response studies in other populations are also needed.

Some limitations of our study should be mentioned. First, studies that reported their effect size as SMD were limited. As a result, subgroup analysis was not performed for these studies. Second, the results of all analyzes showed that zinc has a positive effect on glycemic biomarkers in diabetic patients. However, its results on other diseases that have insulin resistance conditions are not certain and need more studies. Third, our results on FBS should be interpreted with caution due to high heterogeneity. However, it was tried to check the sources of heterogeneity using subgroup analysis. This statistical heterogeneity was due to variability in the intervention effects caused by methodological differences between studies (dose, duration, and mean age of participants). Fourth, the first is the repetition of some studies in different meta-analyses, which can affect the final result. Further evaluations indicated that the repeated studies did not have a significant impact on the final outcome. Along with the limitations, we should also mention the strengths. All systematic reviews of RCTs that investigated the effects of zinc supplementation on glycemic indices were included in the current umbrella review. Most of the included studies were of high quality and low risk of bias. In addition, potential sources of bias were assessed. Also, most subgroup analyzes had low heterogeneity, which indicates acceptable validity of the results. Regarding the overall results, except for FBS-WMD, the rest of the results had low heterogeneity.

## Conclusion

Zinc supplementation has an improving effect on FBS, HbA1c, and HOMA-IR. The significant effect of zinc on insulin was confirmed in SMD analysis. Subgroup analysis based on health condition revealed that diabetic patients benefited more from zinc supplementation to reduce glycemic parameters compared to other health conditions. According to evidence-based recommendations, nutritional supplements are not supported for diabetic patients and instead it is recommended that micronutrients be acquired from a well-balanced diet. However, diabetic patients often suffer from micronutrient deficiencies and may also need supplementation [4]. The results are valid due to the high quality of the included studies and the low heterogeneity of the results.

### Supplementary Information

Supplementary material 1. Search strategy.

## Data Availability

Not applicable.
