# Supplementary material for: Effect of zinc supplementation on glycemic biomarkers: an umbrella of interventional meta-analyses
Source: Diabetol Metab Syndr. 2024 Jun 8;16:124. doi: 10.1186/s13098-024-01366-0 (PMC11162043; doi:10.1186/s13098-024-01366-0)
Supplement: Supplementary file 1 — Supplementary material 1. Search strategy. [file 13098_2024_1366_MOESM1_ESM.docx]

| Intervention | outcome | RCT |
| --- | --- | --- |
| Zinc OR Zn | "Insulin" OR "Glycated Hemoglobin A" OR "hba1c" OR "homa-ir" OR "quantitative insulin sensitivity check index" OR "quicki" OR "Glucose" OR "fasting blood glucose" OR "fbs" OR "fasting blood sugar" OR "fasting plasma glucose" OR "fpg" OR "diabetes" OR "Diabetes Mellitus" OR "hyperglycemia" OR "Insulin Resistance" OR "glycemic" OR "Glycemic Index" OR "blood glucose" OR "Glycemic Control" OR "diabetes mellitus, type 2" OR "T2DM" | "Systematic review"  OR "Meta-analysis" |

**Supplementary Table.1**: The following search strategy using MeSH terms and keywords was applied;

|  | Search strategy |
| --- | --- |
| PubMed | ("Zinc"[Title/Abstract] OR "Zn "[Title/Abstract]) **AND** ("Insulin"[MeSH Terms] OR "Glycated Hemoglobin A"[Title/Abstract] OR "hba1c"[Title/Abstract] OR "homa-ir"[Title/Abstract] OR "quantitative insulin sensitivity check index"[Title/Abstract] OR "quicki"[Title/Abstract] OR "Glucose"[Title/Abstract] OR "fasting blood glucose"[Title/Abstract] OR "fbs"[Title/Abstract] OR "fasting blood sugar"[Title/Abstract] OR "fasting plasma glucose"[Title/Abstract] OR "fpg"[Title/Abstract] OR "diabetes"[Title/Abstract] OR "Diabetes Mellitus"[Title/Abstract] OR "Diabetes Mellitus"[MeSH Terms] OR "hyperglycemia"[Title/Abstract] OR "Insulin Resistance"[Title/Abstract] OR "glycemic"[Title/Abstract] OR "Glycemic Index"[Title/Abstract] OR "blood glucose"[MeSH Terms] OR "Glycemic Control"[Title/Abstract] OR "diabetes mellitus, type 2"[MeSH Terms] OR "diabetes mellitus type 2"[Title/Abstract] OR "T2DM"[Title/Abstract]) **AND** ("systematic review"[Publication Type] OR "Meta-analysis"[Title/Abstract]. |
| Scopus | (TITLE-ABS-KEY ("Zinc" OR "Zn") AND TITLE-ABS-KEY("Insulin" OR "Glycated Hemoglobin A" OR "hba1c" OR "homa-ir" OR "quantitative insulin sensitivity check index" OR "quicki" OR "Glucose" OR "fasting blood glucose" OR "fbs" OR "fasting blood sugar" OR "fasting plasma glucose" OR "fpg" OR "diabetes" OR "Diabetes Mellitus" OR "hyperglycemia" OR "Insulin Resistance" OR "glycemic" OR "Glycemic Index" OR "blood glucose" OR "Glycemic Control" OR "diabetes mellitus, type 2" OR "T2DM") AND TITLE-ABS-KEY("systematic review" OR "Meta-analysis") |
| ISI web of science | [("Zinc" OR "Zn " (Topic) and "Insulin" OR "Glycated Hemoglobin A" OR "hba1c" OR "homa-ir" OR "quantitative insulin sensitivity check index" OR "quicki" OR "Glucose" OR "fasting blood glucose" OR "fbs" OR "fasting blood sugar" OR "fasting plasma glucose" OR "fpg" OR "diabetes" OR "Diabetes Mellitus" OR "hyperglycemia" OR "Insulin Resistance" OR "glycemic" OR "Glycemic Index" OR "blood glucose" OR "Glycemic Control" OR "diabetes mellitus, type 2" OR "T2DM" (Topic) and ("systematic review" OR "Meta-analysis") (Topic)](https://www.webofscience.com/wos/woscc/summary/e675271b-b548-4c52-9110-6c204b8ca95b-4722a71d/relevance/1) |
| EMBASE | 1 zinc.ab, ti.  2 "diabet*".ab,ti.  3 DM.ab, ti.  4 T2M.ab, ti.  5 blood glucose.ab,ti.  6 blood sugar.ab,ti.  7 glycemic.ab, ti.  8 "hyperglycemi*".ab,ti.  9 HbA1c  10 "systematic review*".ab,ti.  11 " Meta-analysis ".ab,ti.  12 2 or 3 or 4  13 5 or 6 or 7 or 8 OR 9  14 10 or 11  15 1 and 12 and 13 and 15 |
| Cochrane | 1 Mesh descriptor: (Zinc supplementation) explode all trees  2 ((Zinc supplementation) or (Zn)): ti, ab, kw  3 Or 1–2  4 Mesh descriptor:(diabet) explode all trees  5 ((diabet) or (DM) or (T2M) or (blood glucose) or (blood sugar) or (glycemic) or (hyperglycemi) or (HbA1c)): ti, ab, kw  6 Or 4–5  7 MeSH descriptor: (systematic review) explode all trees  8 ((systematic review) or (Meta-analysis)): ti, ab, kw  9 Or 7–8  10 3 and 6 and 9 |

Sensitivity analysis

A. The results of sensitivity analysis for FBS based on WMD analysis.

------------------------------------------------------------------------------

Study omitted | Estimate [95% Conf. Interval]

-------------------+----------------------------------------------------------

Ghaedi (2023) | -12.801284 -16.579365 -9.023201

Jayawardena (2012)| -13.388868 -17.318157 -9.4595795

Nazari (a) (2023) | -13.335308 -17.465279 -9.2053375

Nazari (b) (2023) | -14.355515 -18.890724 -9.8203058

Pompano (A) (2021)| -13.265161 -17.183289 -9.3470345

Pompano (B) (2021)| -14.373159 -18.441534 -10.304785

Pompano (C) (2021)| -14.34366 -18.454327 -10.232994

Pompano (D) (2021)| -13.206286 -17.188158 -9.2244167

Wang (2019) | -13.82126 -18.250189 -9.3923311

Wang (2023) | -12.033634 -15.355505 -8.7117624

Yang (2023) | -14.608686 -18.445953 -10.77142

-------------------+----------------------------------------------------------

Combined | -13.575276 -17.376503 -9.7740488

-------------------+----------------------------------------------------------

B. The results of sensitivity analysis for HbA1c based on WMD analysis.

------------------------------------------------------------------------------

Study omitted | Estimate [95% Conf. Interval]

-------------------+----------------------------------------------------------

Ghaedi (2023) | -.33760363 -.42039332 -.25481391

Jayawardena (2012)| -.33949479 -.42031628 -.25867331

Nazari (a) (2023) | -.34211534 -.42554915 -.25868154

Nazari (b) (2023) | -.34485561 -.4314566 -.25825465

Pompano (A) (2021)| -.34953201 -.42927146 -.26979256

Pompano (B) (2021)| -.35011452 -.43072119 -.26950783

Pompano (C) (2021)| -.38567194 -.47056371 -.30078015

Pompano (D) (2021)| -.34834304 -.4276323 -.26905379

Wang (2019) | -.35124528 -.43206677 -.27042383

Wang (2023) | -.34386238 -.42397505 -.26374975

Yang (2023) | -.37488508 -.46202552 -.28774464

-------------------+----------------------------------------------------------

Combined | -.35117277 -.42960531 -.27274022

C. The results of sensitivity analysis for HOMA-IR based on WMD analysis.

------------------------------------------------------------------------------

Study omitted | Estimate [95% Conf. Interval]

-------------------+----------------------------------------------------------

Ghaedi (2023) | -.51603925 -.65738785 -.37469062

Nazari (a) (2023) | -.54371428 -.67515159 -.41227704

Nazari (b) (2023) | -.54235214 -.71551424 -.36919004

Pompano (A) (2021)| -.5156492 -.66729033 -.36400807

Pompano (B) (2021)| -.53458786 -.69442469 -.37475106

Pompano (C) (2021)| -.50977582 -.66463572 -.35491595

Pompano (D) (2021)| -.54011256 -.68505239 -.39517272

Wang (2019) | -.50640446 -.64589596 -.3669129

Wang (2023) | -.49689525 -.61663979 -.37715068

Yang (2023) | -.52354592 -.69559205 -.35149974

-------------------+----------------------------------------------------------

Combined | -.52036134 -.65732658 -.38339609

------------------------------------------------------------------------------

D. The results of sensitivity analysis for insulin based on WMD analysis.

------------------------------------------------------------------------------

Study omitted | Estimate [95% Conf. Interval]

-------------------+----------------------------------------------------------

Ghaedi (2023) | -.25763759 -.86887187 .35359672

Nazari (a) (2023) | -.27553082 -.92663807 .37557644

Nazari (b) (2023) | -.01277282 -.79382277 .76827711

Wang (2019) | -.09736075 -.47119126 .27646977

Wang (2023) | -.18332565 -1.2505407 .88388944

-------------------+----------------------------------------------------------

Combined | -.15728863 -.74003372 .42545645

------------------------------------------------------------------------------


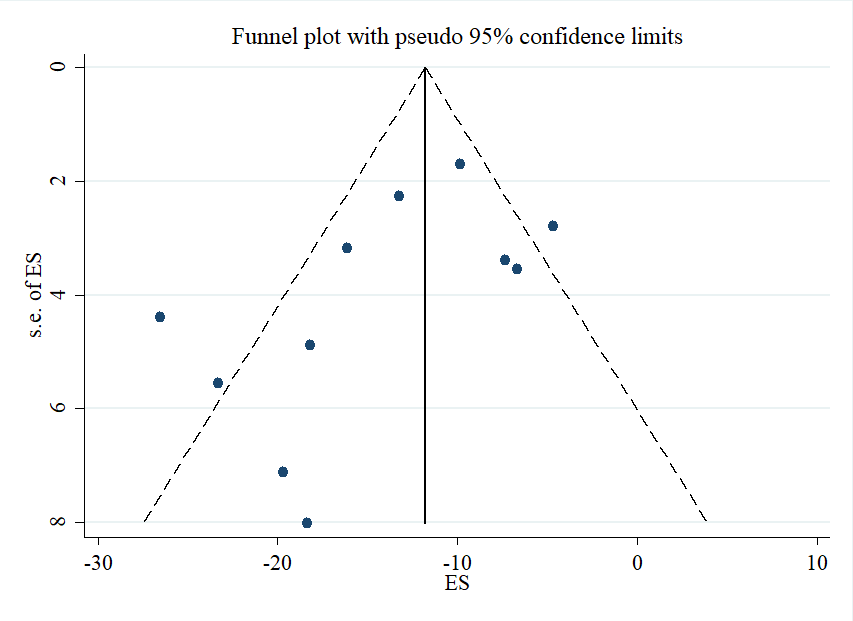


**Figure S1.** Forest plot of the effect of zinc on FBS based on WMD analysis.


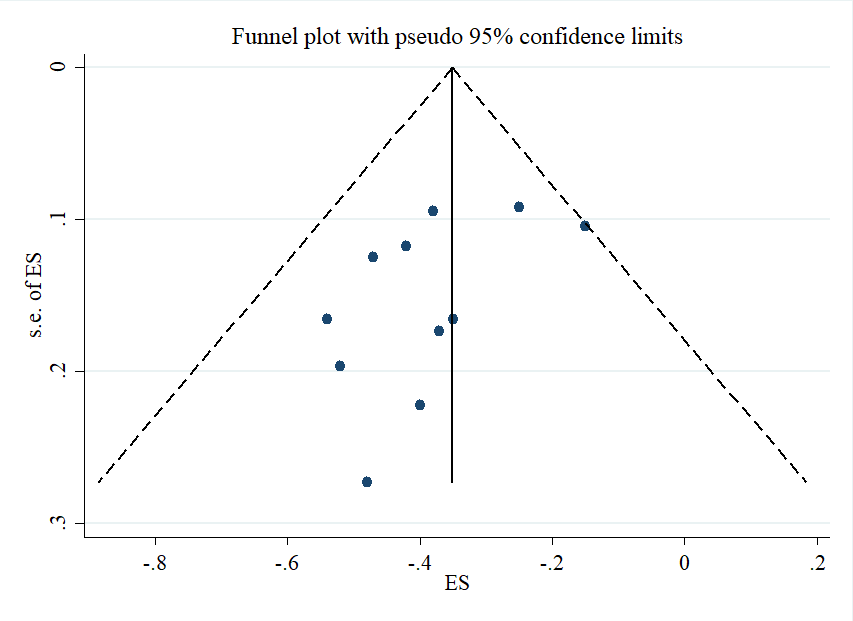


**Figure S2.** Forest plot of the effect of zinc on HbA1c based on WMD analysis.


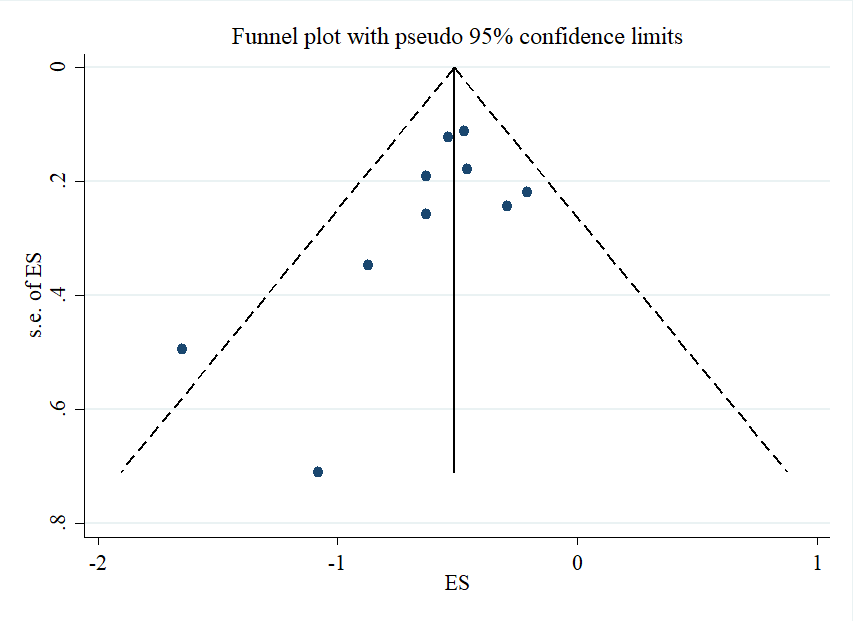


**Figure S3.** Forest plot of the effect of zinc on HOMA-IR based on WMD analysis.


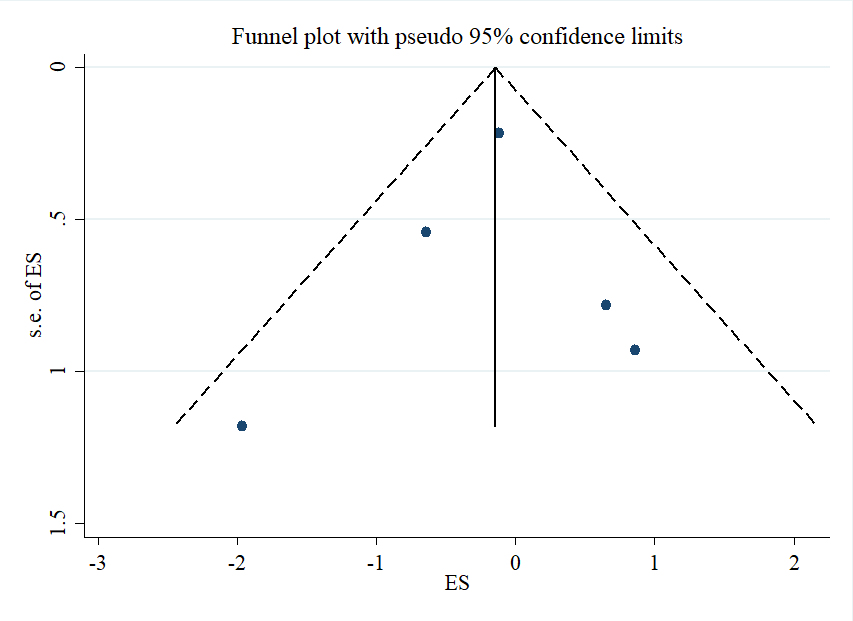


**Figure S4.** Forest plot of the effect of zinc on insulin based on WMD analysis.
